# Malignancy prediction of cutaneous and subcutaneous neoplasms in canines using B-mode ultrasonography, Doppler, and ARFI elastography

**DOI:** 10.1186/s12917-021-03118-y

**Published:** 2022-01-03

**Authors:** Igor Cezar Kniphoff da Cruz, Rafael Kretzer Carneiro, Andrigo Barboza de Nardi, Ricardo Andrés Ramirez Uscategui, Eduarda Mazzardo Bortoluzzi, Marcus Antônio Rossi Feliciano

**Affiliations:** 1 Universidade Estadual Paulista “Júlio de Mesquita Filho”, Via de acesso Professor Paulo Donato Castellane, s/n, Vila Industrial, Jaboticabal, CEP 14884-900 Brazil; 2 Universidad CES, Medellín, Colombia; 3 Universidade Federal do Vale do Jequitinhonha e Mucuri, Unaí, Brazil; 4 Kansas State University, Manhattan, USA; 5 Universidade Federal de Santa Maria, Santa Maria, Brazil

**Keywords:** Oncology, Ultrasonography, Cancer, Canine

## Abstract

**Background:**

Cutaneous and subcutaneous neoplasms are highly prevalent in dogs, ranging from benign to highly aggressive and metastatic lesions. The diagnosis is obtained through histopathology, however it is an invasive technique that may take a long time to obtain the result, delaying the beginning of the adequate treatment. Thus, there is a need for non-invasive tests that can help in the early diagnosis of this type of cancer. The aim of this study was to verify the accuracy of B-mode ultrasonography, Doppler, and ARFI elastography to predict malignancy in cutaneous and subcutaneous canine neoplasms. In addition, we aim to propose an ultrasonography evaluation protocol and perform the neoplasms characterization using these three proposed techniques.

**Results:**

Twenty-one types of specific neoplasm were diagnosed, and using B-mode, we verified the association between heterogeneous echotexture, invasiveness, presence of hyperechoic spots, and cavity areas with malignancy. An increased pulsatility was verified in malignant neoplasms using Doppler (cut-off value > 0.93). When using the elastography, malignancy was associated with non-deformable tissues and shear wave velocity > 3.52 m/s. Evaluation protocols were proposed associating 4, 5, 6, or 7 malignancy predictive characteristics, and characterization was done for all tumors with at least two cases.

**Conclusions:**

We concluded that ultrasonography methods are promising and effective in predicting malignancy in these types of tumors, and the association of methods can increase the specificity of the results.

**Supplementary Information:**

The online version contains supplementary material available at 10.1186/s12917-021-03118-y.

## Background

The cutaneous and subcutaneous neoplasms are frequently observed in the canine species, originating from different cell types, and may present different biological behaviors from benign to highly aggressive and metastatic lesions [1, 2]. These lesions, especially the malignant ones, can promote significant alterations and can cause pain, inflammation, infections, hemodynamic changes, and when metastatic can compromise the function of other organs, which can lead to death [3, 4]. Fast and accurate diagnosis is essential to establish adequate therapy favoring the prognosis and survival of patients [5, 6].

The final diagnosis is obtained via histopathologic analysis, whose samples were collected by incisional or excisional biopsies. Both biopsy techniques are invasive and require anesthesia for the patients [7]. In many cases, there is a delay in obtaining the results, that may slow down the therapeutic approach, which is essential to increase patient survival [8].

The cytopathological evaluation can be used to provide faster results besides being more cost-efficient. However, cytopathology is an invasive technique that cannot promote the final diagnosis, grade some tumor types, have a considerable rate of inconclusive results, and its diagnostic accuracy is variable [9–11]. When considering the limitations of this type of evaluation, fast and noninvasive techniques are required to aid in early diagnosis and therapeutic management.

In human medicine, the ultrasonography technique has been used since the 1990s in skin cancer studies, and it already has applicability in malignancy prediction and differentiation between some tumor types, such as squamous cells and basal cell carcinomas [12, 13]. In veterinary medicine, specifically in the canine species, B-mode, Doppler, and ARFI elastography have already shown promise in malignancy prediction of breast tumors, where malignant neoplasms presented increased dimension and higher systolic and diastolic vascular velocities, as well as high shear velocity values (> 2.57 m/s) [14].

As for cutaneous and subcutaneous canine neoplasms, a few studies have demonstrated the applicability of ultrasonographic methods on tumor diagnosis and differentiation. A preliminary study found that predominantly hypoechoic, heterogeneous neoplasms with signs of invasiveness in adjacent tissues were more prone to malignancy [15]. On Doppler, it has been verified that cutaneous mast cell tumors have lower resistivity indices than soft tissues sarcomas and benign lesions [16]. Malignant neoplasms demonstrated greater stiffness on elastography when compared to benign tissues. However, the assessment was only qualitative, without the obtention of quantitative values of this stiffness [17].

Based on the possibility of skin tumors malignancy prediction in canines using ultrasound techniques, this study aimed to evaluate cutaneous and subcutaneous neoplasms using B-mode, Doppler, and ARFI elastography, to determine the accuracy of ultrasonography methods, suggest an evaluation protocol for these neoplasms, and perform the ultrasonographic characterization of the specific tumor types included.

## Results

### Histopathologic results

A total of 130 cutaneous and subcutaneous neoplasms (98 malignant and 32 benign) were evaluated, resulting in 21 histopathologic classifications (Table 1). The most prevalent malignant neoplasms in this study were the high-grade cutaneous mast cell tumors (18.46%). In comparison, the most prevalent benign neoplasms were lipomas (13.07%).

**Table 1:** Histopathologic classification, malignancy, and number of cutaneous and subcutaneous neoplasms evaluated by ultrasound

Classification	Malignancy	n
Adenocarcinoma	Malignant	1
Sebaceous adenoma	Benign	6
Basal cell carcionoma	Malignant	2
Squamous cell carcinoma	Malignant	15
Mixed carcinoma	Malignant	2
Apocrine cystadenoma	Benign	2
Fibrosarcoma	Malignant	4
Cavernous hemangioma	Benign	6
Hemangiopericytoma	Malignant	1
Hemangiosarcoma	Malignant	5
Cutaneous lymphoma	Malignant	13
Lipoma	Benign	17
Infiltrative lipoma	Benign	1
High-grade cutaneous mast cell tumor	Malignant	24
Low-grade cutaneous mast cell tumor	Malignant	10
Combined subcutaneous mast cell tumor	Malignant	1
Infiltrative subcutaneous mast cell tumor	Malignant	2
Amelanotic melanoma	Malignant	9
Grade II soft tissue sarcoma	Malignant	7
Grade III soft tissue sarcoma	Malignant	1
Grade II perivascular sheath tumor	Malignant	1
**Total**	**–**	**130**

### B-mode ultrasonography

**Fig. 1:**
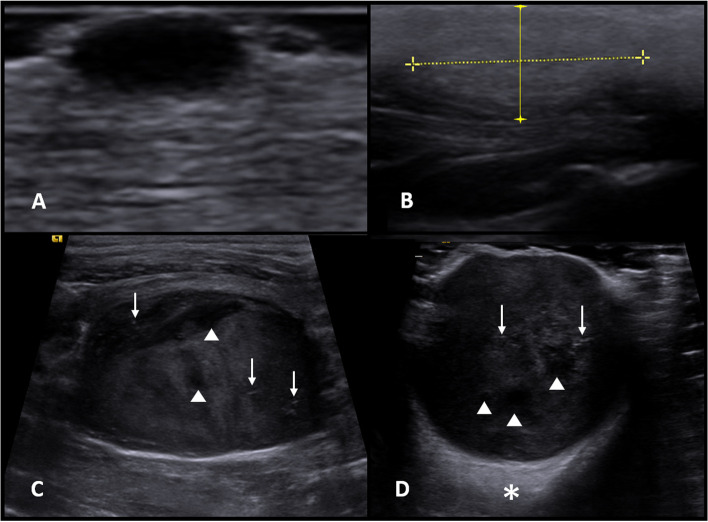
B-mode ultrasound images in longitudinal section of canine cutaneous neoplasms. **a** Apocrine cystadenoma - predominantly hypoechoic, homogeneous, non-encapsulated, noninvasive, lacking hyperechogenic spots and cavitary areas; **b** lipoma - predominantly hyperechogenic, homogeneous, non-encapsulated, noninvasive, with absence of hyperechogenic points and cavitary areas; **c** Low-grade mast cell tumor - predominantly hypoechogenic, heterogeneous, partially encapsulated, with signs of invasiveness in adjacent tissues, presence of hyperechogenic spots (arrows), and cavitary areas (arrowheads); **d** high-grade mast cell tumor - predominantly hypoechogenic, heterogeneous, encapsulated, with signs of invasiveness in adjacent tissues, presence of hyperechogenic spots (arrows), and cavitary areas (arrowheads). Note reactivity of adjacent musculature (asterisk)

In B-mode, measurements of length (3.02 ± 2.85 cm), width (2.58 ± 2.18 cm), and height (1.79 ± 1.71 cm) were not associated with tumor malignancy, as well as echogenicity, capsule, and echotexture pattern (smooth or rough) (Table 2). It was found that echotexture (*P* = 0.007), invasiveness in adjacent tissues (*P* = 0.002), hyperechogenic spots (*P* = 0.031), and cavitary areas (*P* = 0.001) were shown to be predictive characteristics of malignancy. This way, heterogeneous neoplasms with signs of invasiveness, presence of hyperechogenic spots, and cavitary areas were more likely to be malignant (Fig. 1). The predictive values ​​of sensitivity, specificity, accuracy, PPV and NPV are shown in Table 2, and the ultrasonographic characterization of neoplasms is shown in Table 3.

**Table 2:** Results of association between the mode-B ultrasonographic characterization of malignant cutaneous and subcutaneous canine neoplasms and their predictive values (sensibility, specificity, accuracy, positive predictive value, and negative predictive value) for those with *P* < 0.05

Characteristic	***P***-value	Se (%)	Sp (%)	Ac (%)	PPV (%)	NPV (%)
Length	0.780	–	–	–	–	–
Width	0.795	–	–	–	–	–
Height	0.619	–	–	–	–	–
Echogenicity	0.059	–	–	–	–	–
Echotexture	0.007	96.87	18.75	93.04	78.15	66.66
Echotexture pattern	0.915	–	–	–	–	–
Invasiveness	0.002	70.83	59.37	68.14	83.95	40.42
Capsule	0.099	–	–	–	–	–
Hyperechoic spots	0.031	50.00	71.87	55.38	84.48	31.94
Cavity areas	0.001	69.38	62.50	67.69	85.00	40.00

*Se* sensitivity; *Sp* specificity; *Ac* accuracy; *PPV* positive predictive value; *NPV* negative predictive value

**Table 3:** B-mode ultrasonographic characterization (echogenicity, echotexture, echotexture pattern, invasiveness, capsule, hyperechogenic spots, and cavitary areas) of cutaneous and subcutaneous canine neoplasms for tumor types that presented two or more cases

Histopathological classification (n)	Echogenicity	Echotexture	Echotexture pattern	Invasiveness	Capsule	Hyperechogenic spots	Cavitary areas
Sebaceous adenoma (6)	Hypoechogenic 83.3% Hyperechogenic 16.7%	Homogeneous16.7% Heterogeneous 83.3%	Smooth 33.3% Gross 66.7%	Invasive 50% Noninvasive 50%	Present 16.7% Absent 83.3%	Present 50% Absent 50%	Present 50% Absent 50%
Basal cell carcinoma (2)	Hypoechogenic 50% Hyperechogenic 50%	Homogeneous 0% Heterogeneous 100%	Smooth 0% Gross 100%	Invasive 100% Noninvasive 0%	Present 50% Absent 50%	Present 100% Absent 0%	Present 100% Absent 0%
Squamous cell carcinoma (15)	Hypoechogenic 53.3% Hyperechogenic 46.7%	Homogeneous 0% Heterogeneous 100%	Smooth 60% Gross 40%	Invasive 66.7% Noninvasive 33.3%	Present 6.7% Absent 93.3%	Present 40% Absent 60%	Present 60% Absent 40%
Mixed carcinoma (2)	Hypoechogenic 100% Hyperechogenic 0%	Homogeneous 0% Heterogeneous 100%	Smooth 0% Gross 100%	Invasive 100% Noninvasive 0%	Present 0% Absent 100%	Present 0% Absent 100%	Present 0% Absent 100%
Apocrine cystadenoma (2)	Hypoechogenic 100% Hyperechogenic 0%	Homogeneous 50% Heterogeneous 50%	Smooth 50% Gross 50%	Invasive 50% Noninvasive 50%	Present 0% Absent 100%	Present 0% Absent 100%	Present 50% Absent 50%
Fibrosarcoma (4)	Hypoechogenic 100% Hyperechogenic 0%	Homogeneous 0% Heterogeneous 100%	Smooth 25% Gross 75%	Invasive 75% Noninvasive 25%	Present 25% Absent 75%	Present 50% Absent 50%	Present 70% Absent 30%
Cavernous hemangioma (6)	Hypoechogenic 100% Hyperechogenic 0%	Homogeneous 33.3% Heterogeneous 66.7%	Smooth 83.3% Gross 16.7%	Invasive 16.7% Noninvasive 83.3%	Present 33.3% Absent 67.7%	Present 33.3% Absent 67.7%	Present 50% Absent 50%
Hemangiosarcoma (5)	Hypoechogenic 100% Hyperechogenic 0%	Homogeneous 0% Heterogeneous 100%	Smooth 80% Gross 20%	Invasive 20% Noninvasive 80%	Present 0% Absent 100%	Present 20% Absent 80%	Present 100% Absent 0%
Cutaneous lymphoma (13)	Hypoechogenic 84.6% Hyperechogenic 15.4%	Homogeneous 0% Heterogeneous 100%	Smooth 61.5% Gross 38.5%	Invasive 100% Noninvasive 0%	Present 15.4% Absent 84.6%	Present 15.4% Absent 84.6%	Present 46.2% Absent 53.8%
Lipoma (17)	Hypoechogenic 41.2% Hyperechogenic 58.8%	Homogeneous 11.8% Heterogeneous 88.2%	Smooth 41.2% Gross 58.8%	Invasive 41.2% Noninvasive 58.8%	Present 0% Absent 100%	Present 17.6% Absent 82.4%	Present 83.5% Absent 16.5%
High-grade cutaneous mast cell tumor (24)	Hypoechogenic 79.2% Hyperechogenic 20.8%	Homogeneous 4.2% Heterogeneous 95.8%	Smooth 45.8% Gross 54.2%	Invasive 79.2% Noninvasive 20.8%	Present 29.2% Absent 70.8%	Present 58.3% Absent 41.7%	Present 83.3% Absent 16.7%
Low-grade cutaneous mast cell tumor (10)	Hypoechogenic 90% Hyperechogenic 10%	Homogeneous 0% Heterogeneous 100%	Smooth 20% Gross 80%	Invasive 80% Noninvasive 20%	Present 20% Absent 80%	Present 90% Absent 10%	Present 90% Absent 10%
Infiltrative subcutaneous mast cell tumor (2)	Hypoechogenic 100% Hyperechogenic 0%	Homogeneous 0% Heterogeneous 100%	Smooth 0% Gross 100%	Invasive 100% Noninvasive 0%	Present 100% Absent 0%	Present 0% Absent 100%	Present 0% Absent 100%
Amelanotic melanoma (9)	Hypoechogenic 100% Hyperechogenic 0%	Homogeneous 0% Heterogeneous 100%	Smooth 33.3% Gross 66.7%	Invasive 50% Noninvasive 50%	Present 16.7% Absent 83.3%	Present 44.4% Absent 55.6%	Present 55.6% Absent 44.4%
Grade II soft tissue sarcoma (7)	Hypoechogenic 100% Hyperechogenic 0%	Homogeneous 0% Heterogeneous 100%	Smooth 100% Gross 0%	Invasive 100% Noninvasive 0%	Present 100% Absent 0%	Present 100% Absent 0%	Present 100% Absent 0%

### Doppler

The absence of vascularization on Doppler was verified in 59 neoplasms, while 39 had mild vascularization, 21 moderate, and 11 intense. Although an association between vascularization intensity and malignancy was not observed (Table 4), only one benign neoplasm (infiltrative lipoma) presented intense vascularization. There were also no associations between tumor malignancy, location, and vascularization pattern.

**Table 4:** Results of the association between the characteristics observed by color Doppler and pulsed Doppler with malignant cutaneous and subcutaneous canine neoplasms and their predictive values (cut-off value, sensibility, specificity, and area under the curve) for those with *P* < 0.05

Characteristic	***P***-value	Cut-off value	Se (%)	Sp (%)	AUC (%)
Intensity	0.211	–	–	–	–
Location	0.617	–	–	–	–
Patter	0.171	–	–	–	–
Systolic peak	0.635	–	–	–	–
Diastolic velocity	0.971	–	–	–	–
Resistivity index	0.071	–	–	–	–
Pulsatility index	0.015	> 0.93	90.50	55.60	75.70

*Se* sensitivity; *Sp* specificity; *AUC* area under the curve

**Fig. 2:**
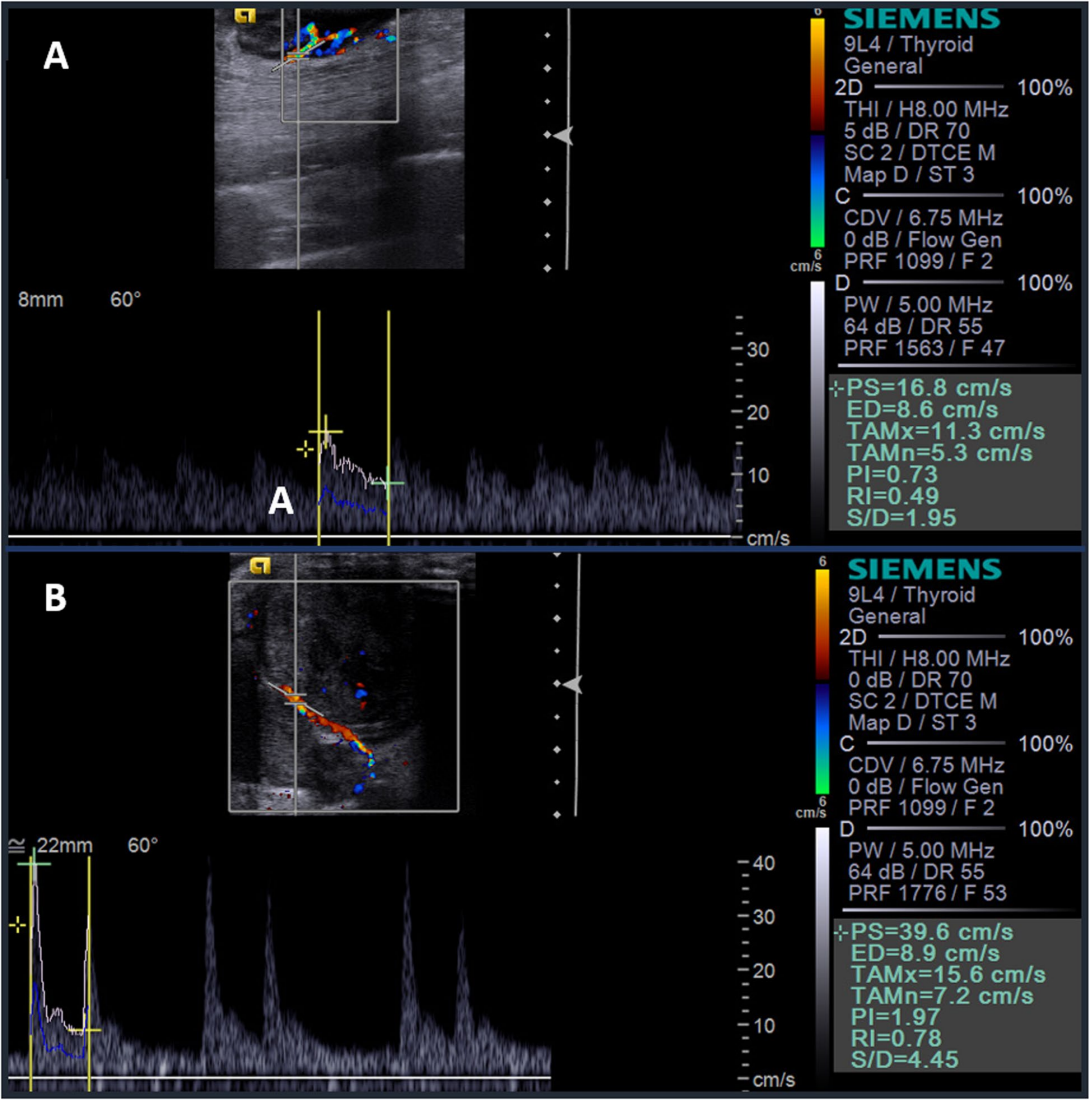
Ultrasonographic images of canine cutaneous neoplasms obtained by pulsed Doppler, showing spectral tracings and Doppler velocimetry indices calculations. **a** Benign neoplasm (cavernous hemangioma) with 0.73 (not indicative of malignancy) pulsatility index (PI); **b** Malignant neoplasm (grade II soft tissue sarcoma) presenting 1.97 PI (indicating malignancy)

Identification of arterial flow using pulsed Doppler was only possible in 51 neoplasms. Of these, 42 were malignant (82.35%) and only nine benign (17.65%). It was found that the peak values ​​of systolic velocity, diastolic velocity, and resistivity index were not predictive of malignancy using the pulsed Doppler. However, the pulsatility index proved to be significant in differentiating between malignant and benign neoplasms (*P* = 0.015), with a cut-off value > 0.93 as indicative of malignancy, with 90.5% sensitivity, 55.6% specificity, and 75.7% accuracy (Fig. 2). The Doppler ultrasonographic characterization of cutaneous neoplasms is shown in Table 5.

**Table 5:** Ultrasonographic characterization by Doppler (intensity, location and vascularization pattern, systolic peak, diastolic velocity, resistivity index, and pulsatility index) of cutaneous and subcutaneous canine neoplasms for tumor types that presented two or more cases

Histopathological classification (n)	Intensity	Location	Pattern	SP (cm/s) (mean ± SD)	DV (cm/s) (mean ± SD)	RI (mean ± SD)	PI (mean ± SD)
Sebaceous adenoma (6)	Absent 33.3% Discrete 16.7% Moderate 50% Intense 0%	Central 0% Peripheral 0% Diffuse 100%	Perinodular 0% Mosaic 25% Network 75%	8.4 ± 2.21	2.8 ± 1.22	0.67 ± 0.12	2.03 ± 1.14
Basal cell carcinoma (2)	Absent 0% Discrete 100% Moderate 0% Intense 0%	Central 0% Peripheral 0% Diffuse 100%	Perinodular 0% Mosaic 0% Network 100%	16.73 ± 29.18	4.45 ± 5.88	0.7 ± 0.11	5.0 ± 9.32
Squamous cell carcinoma (15)	Absent 80% Discrete 13.3% Moderate 0% Intense 6.7%	Central 0% Peripheral 0% Diffuse 100%	Perinodular 0% Mosaic 66.7% Network 33.3%	10.15 ± 8.55	5.5 ± 5.65	0.52 ± 0.15	1.52 ± 0.91
Mixed carcinoma (2)	Absent 100% Discrete 0% Moderate 0% Intense 0%	NA	NA	NA	NA	NA	NA
Apocrine cystadenoma (2)	Absent 50% Discrete 50% Moderate 0% Intense 0%	Central 0% Peripheral 100% Diffuse 0%	Perinodular 100% Mosaic 0% Network 0%	NA	NA	NA	NA
Fibrosarcoma (4)	Absent 75% Discrete 25% Moderate 0% Intense 0%	Central 0% Peripheral 50% Diffuse 50%	Perinodular 0% Mosaic 100% Network 0%	NA	NA	NA	NA
Cavernous hemangioma (6)	Absent 50% Discrete 50% Moderate Intense	Central 0% Peripheral 33.3% Diffuse 66.7%	Perinodular 0% Mosaic 100% Network 0%	5.85 ± 1.77	2.35 ± 0.35	0.58 ± 0.06	1.06 ± 0.17
Hemangiosarcoma (5)	Absent 80% Discrete 20% Moderate 0% Intense 0%	Central 0% Peripheral 0% Diffuse 100%	Perinodular 0% Mosaic 100% Network 0%	9.2*	3.4*	0.63*	4.14*
Cutaneous lymphoma (13)	Absent 38.5% Discrete 53.8% Moderate 7.7% Intense 0%	Central 0% Peripheral 62.5% Diffuse 37.5%	Perinodular 62.5% Mosaic 37.5% Network 0%	64.7 ± 40.91	18.8 ± 17.16	0.73 ± 0.18	2.18 ± 1.35
Lipoma (17)	Absent 70.6% Discrete 23.5% Moderate 5.9% Intense 0%	Central 20% Peripheral 20% Diffuse 60%	Perinodular 0% Mosaic 40% Network 60%	18.03 ± 5.71	8.67 ± 1.24	0.49 ± 1.74	0.82 ± 0.58
High-grade cutaneous mast cell tumor (24)	Absent 4.2% Discrete 33.3% Moderate 41.7% Intense 20.8%	Central 13% Peripheral 8.7% Diffuse 78.3%	Perinodular 4.4% Mosaic 39.1% Network 56.5%	17.07 ± 23.89	5.02 ± 5.18	0.66 ± 0.12	3.87 ± 7.2
Low-grade cutaneous mast cell tumor (10)	Absent 40% Discrete 40% Moderate 20% Intense 0%	Central 16.7% Peripheral 33.3% Diffuse 50%	Perinodular 0% Mosaic 66.7% Network 33.3%	28.76 ± 40.17	4.35 ± 5.56	0.67 ± 0.1	4.47 ± 8.83
Infiltrative subcutaneous mast cell tumor (2)	Absent 0% Discrete 0% Moderate 100% Intense 0%	Central 100% Peripheral 0% Diffuse 0%	Perinodular 0% Mosaic 0% Network 100%	2.31 ± 1.12	5.0 ± 4.21	0.78 ± 0.1	4.11 ± 2.32
Amelanotic melanoma (9)	Absent 33.3% Discrete 16.7% Moderate 0% Intense 50%	Central 0% Peripheral 0% Diffuse 100%	Perinodular 0% Mosaic 25% Network 75%	18.55 ± 27.51	4.89 ± 5.81	0.69 ± 0.11	4.29 ± 7.89
Grade II soft tissue sarcoma (7)	Absent 0% Discrete 0% Moderate 50% Intense 50%	Central 0% Peripheral 0% Diffuse 100%	Perinodular 0% Mosaic 0% Network 100%	8.98 ± 6.56	3.37 ± 2.59	0.66 ± 0.1	4.57 ± 8.44

*Only one neoplasm with the arterial flow; *SP* systolic peak; *DV* diastolic velocity; *RI* resistivity index; *PI* pulsatility index; *SD* Standard deviation; *NA* not applicable

### ARFI Elastography

Both qualitative and quantitative assessments were shown to be significant in predicting malignancy (Table 6). Regarding deformability, it was observed that 11 benign and nine malignant neoplasms were classified as deformable, while 21 benign and 89 malignant were non-deformable. Deformability was shown to be predictive of tumor malignancy with 90.2% sensitivity, 35.48% specificity, 87.09% accuracy, 81.3% PPV, and 55% NPV.

**Table 6:** Results of the association between ARFI electrographic findings and malignancy of canine cutaneous and subcutaneous neoplasms

Characteristic	***P***-value	Se (%)	Sp (%)	Ac (%)	PPV (%)	NPV (%)	AUC (%)
Deformability	< 0.001	90.2	35.48	87.09	81.3	55.00	–
SWV	0.024	54.1	68.7	–	–	–	62.7

*SWV* shear wave velocity; *Se* sensitivity; *Sp* specificity; *Ac* accuracy; *PPV* positive predictive value; *NPV* negative predictive value; *AUC* area under the curve

**Fig. 3:**
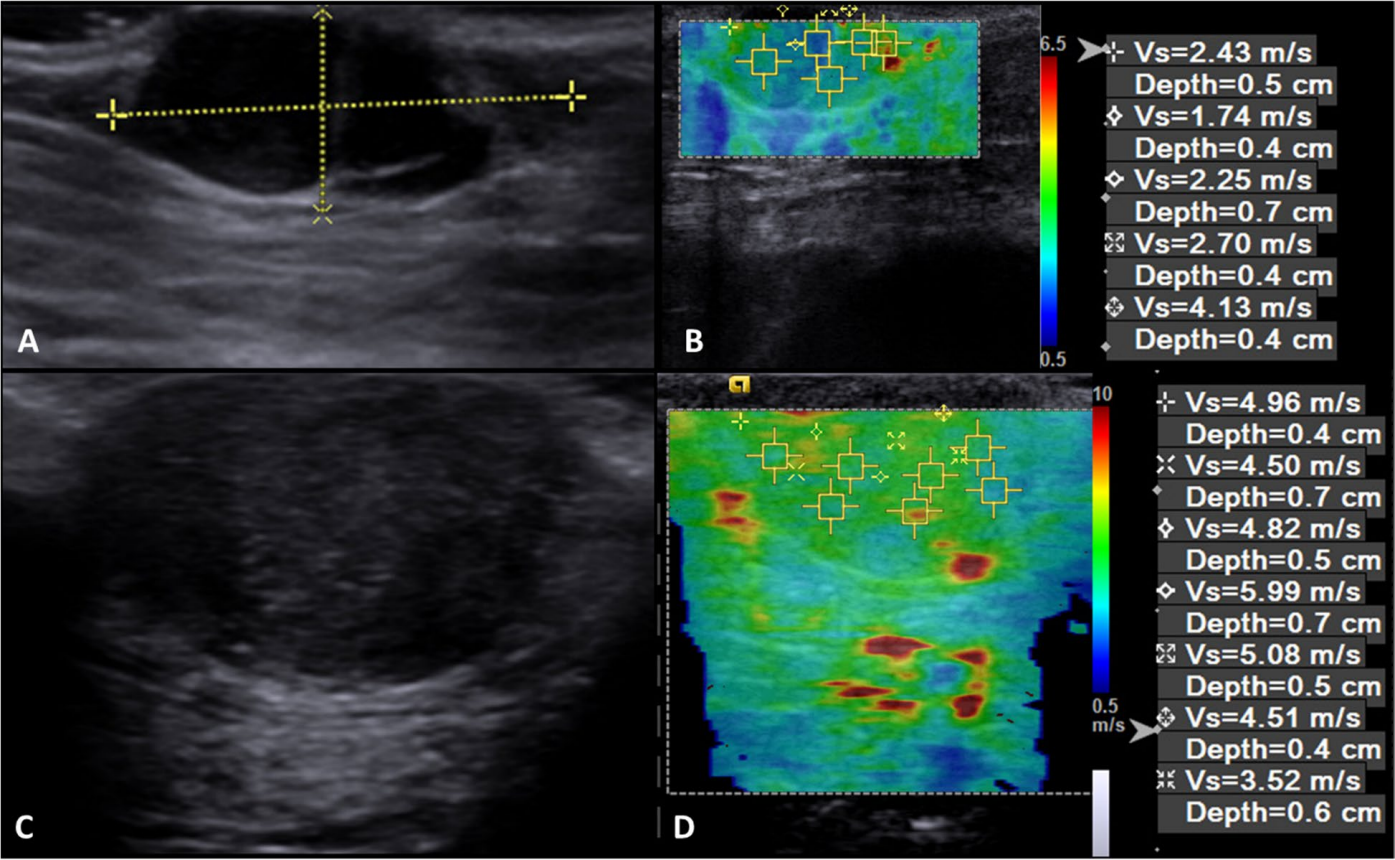
B-mode **(a, c)** and ARFI elastography **(b, d)** ultrasound images of canine cutaneous neoplasms. In **(a)** and **(b)**, hemangioma – elastogram image showing predominance of blue colors (less rigid) and with mean shear wave velocity (SWV) of 2.65 m/s (not indicative of malignancy); In **(c)** and **(d)**, high-grade cutaneous mast cell tumor – elastogram showing a predominance of green and yellow colors (intermediate stiffness) and with an average SWV of 4.77 m/s (indicating malignancy)

In the quantitative elastography study, greater rigidity was observed in malignant (3.72 ± 1.94 m/s) compared to benign neoplasms (3.21 ± 1.86 m/s); consequently, SWV above 3.52 m/s was indicative of malignancy (Fig. 3), with 54.1% sensitivity, 68.7% specificity, and AUC of 62.7%. Among the benign neoplasms, adenomas had high rigidity (4.12 ± 2.06), hence the most rigid adenoma had a mean SWV of 8.3 m/s. Of the malignant neoplasms, the most rigid were soft tissue sarcomas (4.11 ± 1.81 m/s) and mast cell tumors (3.76 ± 1.92 m/s), however, the largest SWV observed was in a squamous cell carcinoma (9.1 m/s). The characterization of tumor stiffness by ARFI elastography is shown in Table 7.

**Table 7:** Ultrasonographic characterization by ARFI elastography (deformability and shear wave velocity – SWV) of cutaneous and subcutaneous canine neoplasms for tumor types that presented two or more cases

Histopathological classification (n)	Deformability	SWV (m/s) (mean ± SD)
Sebaceous adenoma (6)	Deformable 16.7% Non-deformable 83.3%	3.82 ± 1.92
Basal cell carcinoma (2)	Deformable 0% Non-deformable 100%	3.87 ± 1.84
Squamous cell carcinoma (15)	Deformable 0% Non-deformable 100%	3.82 ± 1.96
Mixed carcinoma (2)	Deformable 0% Non-deformable 100%	2.46 ± 0.19
Apocrine cystadenoma (2)	Deformable 0% Non-deformable 100%	3.72 ± 1.81
Fibrosarcoma (4)	Deformable 0% Non-deformable 100%	3.89 ± 1.88
Cavernous hemangioma (6)	Deformable 0% Non-deformable 100%	3.87 ± 1.79
Hemangiosarcoma (5)	Deformable 20% Non-deformable 80%	2.9 ± 1.97
Cutaneous lymphoma (13)	Deformable 0% Non-deformable 100%	3.6 ± 1.97
Lipoma (17)	Deformable 64.7% Non-deformable 35.3%	3.83 ± 1.84
High-grade cutaneous mast cell tumor (24)	Deformable 4.2% Non-deformable 95.3%	3.76 ± 1.92
Low-grade cutaneous mast cell tumor (10)	Deformable 0% Non-deformable 100%	3.91 ± 1.79
Infiltrative subcutaneous mast cell tumor (2)	Deformable 0% Non-deformable 100%	3.96 ± 1.83
Amelanotic melanoma (9)	Deformable 0% Non-deformable 100%	3.72 ± 1.83
Grade II soft tissue sarcoma (7)	Deformable 0% Non-deformable 100%	3.67 ± 1.8

### Association of malignancy predictive characteristics

All characteristics that presented significant results in malignancy prediction were considered for the association between the different types of ultrasound techniques. Thus, seven tumor characteristics were considered: heterogeneous, invasive, presence of hyperechogenic points, presence of cavitary areas, PI above 0.93, non-deformable, and above 3.52 m/s SWV.

It was observed that 85 neoplasms had at least four malignancy predictive characteristics (Table 8). Seventy-two (84.7%) neoplasms were indeed malignant, and only 13 (15.3%) were benign. Five or more malignancy predictive characteristics were found in sixty neoplasms, where 53 (88.3%) were malignant and seven (11.7%) were benign. Forty-five neoplasms had at least six characteristics, where 41 (87.2%) were malignant, and four (12.8%) were benign. When considering all seven malignancy predictive characteristics, 16 neoplasms were computed, where 14 (87.5%) were malignant, and only two (12.5%) were benign.

**Table 8:** Descriptive and predictive values of ultrasound assessment protocols, associating malignancy predictive characteristics of cutaneous and subcutaneous canine neoplasms verified by B-mode ultrasonography, Doppler, and ARFI elastography

Predictive characteristics	Total (n)	Malignant (n)	Benign (n)	***P***-value	Se (%)	Sp (%)	Ac (%)	PPV (%)	NPV (%)
Four or more	85	72	13	0.001	73.46	59.37	70	84.7	42.22
Five or more	60	53	7	0.002	54.08	78.12	60	88.33	35.71
Six or more	45	41	4	0.002	41.83	87.5	53	91.11	32.94
All seven	16	14	2	0.230	–	–	–	–	–

*n* total number; *Se* sensitivity; *Sp* specificity; *Ac* accuracy; *PPV* positive pr edictive value; *NPV* negative predictive value

## Discussion

This study provides important information regarding the diagnosis and classification of cutaneous and subcutaneous canine neoplasms, as it was possible to establish malignancy predictive characteristics by all techniques used (B-mode, Doppler, and ARFI elastography). In addition, it was possible to determine an ultrasound examination protocol that could contribute to lesions diagnosis and prognosis and provide individual ultrasound characteristics for each studied tumor type. Because it is a complementary method, its characteristics are highly sensitive and have a positive predictive value. These results were obtained in all three ultrasound techniques that were performed.

Given the high number of cutaneous and subcutaneous neoplasm types, it should be considered that they have different structural components and biological behaviors. They can range from benign to highly aggressive and metastatic lesions [18], which justifies the moderate results observed. The authors would like to emphasize the importance of studies regarding specific cancer types, as the present study results differed from previous canine mammary tumors findings. In another study, with breast tumors, different characteristics and predictive values ​​of malignancy were found [14].

There were no associations between malignancy and tumor measurements in this study, which can be explained by the fact that neoplasms were diagnosed at different stages. There were no associations with echogenicity, which may be related to the different pathological processes involved, such as active inflammation or tissue necrosis in different tumor types [19]. A preliminary study involving 42 cutaneous neoplasms showed an association between malignancy and hypoechogenicity [15]. A greater number of neoplasms and specific types of skin cancer that were included in the present study may explain the discrepancy between the two studies.

The heterogeneous echotexture indicative of malignancy seen in cutaneous and subcutaneous tumors is explained by the different structural components, such as the presence of cavitary areas, points of fibrosis, or microcalcifications. The association between heterogeneous echotexture and malignancy was already demonstrated in previous studies with different types of neoplasms (cutaneous and mammary) in both humans and animals [13, 15–17, 20].

It was possible to identify the signs of invasiveness in adjacent tissues because of their reactivity or the difficult tumors delimitation and then associate it with malignancy. This association is justified because malignant neoplasms tend to be more aggressive and invasive than benign ones, even requiring a greater safety margin when surgically removed [21].

On Doppler, it was not verified any qualitative characteristic with malignancy. It is known that tumor growth, both in malignant and benign lesions, is dependent on the blood supply [22]. Therefore, it is reasonable the fact that no significant results were obtained in neoplasm differentiation through these characteristics even though other researchers showed associations with malignancy in other tumor types, such as breast cancer in women and canine mast cell tumors [23, 24].

Even though no vascularization points were observed in some tumors by color Doppler, the lack of vascularization should not be ruled out. It is known that the color Doppler technique has limitations at microvascular level and tissue perfusion, requiring other methods for diagnostic complementation, such as contrasted ultrasound [14]. Nevertheless, this technique was not available and could not be tested in the present study. This Doppler technique limitation contributed to the impossibility of evaluating all neoplasms by pulsed Doppler, with the Doppler velocimetry indices being calculated for only a portion of those who presented vascularization in color Doppler.

The lack of association between RI, systolic peak, and diastolic velocity with malignancy could be because it was only possible to identify the arterial flow in 9 benign neoplasms, predominantly in malignant lesions (82.35% of cases). However, a PI increase in malignant neoplasms was verified. The increase in this index has already been associated with malignancy in other types of lesions, such as ovarian and thyroid tumors in humans and metastases in canine lymph nodes. These may be related to the compressive effect tumor, the angiogenesis process, and the presence of arteriovenous shunts, which promote turbulent flows with high perfusion rates [25–27].

In the same way, as B-mode observed heterogeneity, the increased rigidity observed in malignant neoplasms can also be explained by tissue components they may present. In a previous study, greater stiffness was found in malignant mammary tumors in female dogs compared to benign ones, and this increase in stiffness was justified by the presence of areas of fibrosis, microcalcifications, and even collagen deposition [14].

The study of the rigidity of skin neoplasms in dogs has already been carried out qualitatively and semiquantitatively (through scores) through elastography, with greater rigidity being observed in malignant tissues, however no real quantitative values ​​of the shear wave velocity were obtained. Only subjective analysis [17]. On the other hand, this study provides more detailed information regarding neoplasms stiffness since it was possible to verify that an SWV greater than 3.52 m/s was indicative of malignancy. In addition, the elastography method used (ARFI method) allows more reliable results that are easy to perform, with greater reproducibility and less interobserver variability than sonoelastography [28].

Some benign neoplasms, such as adenomas, showed high tissue stiffness, justified by the accumulation of keratin and predominantly lymphoplasmacytic inflammatory infiltrate [29], that cause rigidity alterations in the keratinocytes and extracellular matrix [30, 31].

Because ultrasonography is a complementary exam and should not be used alone to diagnose neoplasms, in this study, we demonstrate the importance of the association between the findings of the different techniques performed. These have been already described for evaluating breast tumors in women, where an increase in accuracy was found when elastography and Doppler findings were associated [24]. In our study, as we increased the number of malignancy predictive characteristics, there was a decrease in the number of false positives, increase in protocol specificity, and positive predictive value.

Among the study’s limitations, it should be considered that some tumor types had a low experimental number, and as noted in this discussion and we had some values ​​discrepancies (e.g., adenomas), which may be responsible for the low specificity and accuracy values that were ​​observed.

## Conclusions

Findings from this study indicate that ultrasonography has good applicability in the malignancy prediction of cutaneous and subcutaneous canine neoplasms through different techniques, so that heterogeneous, invasive neoplasms, with the presence of hyperechogenic points and cavitary areas, with PI greater than 0.93, non-deformable and with SWV greater than 3.52 m/s were more prone to malignancy. This study presents quick and noninvasive results and can be used as a complementary method for this diagnosis. Furthermore, we found that the assessment protocol by associating the findings of different ultrasound techniques allows for greater reliability in diagnosing malignancy in this type of cancer, increasing the specificity according to the greater number of predictive characteristics presented by the neoplasm.

## Methods

### Experimental design

This study was carried out according to the ARRIVE guidelines 2.0 (2020). Prospective data collection was conducted between September 2019 and June 2021. Sixty-six dogs of different breeds and ages (9.45 ± 2.58 years) from the hospital routine presented cutaneous or subcutaneous neoplasms were enrolled in the study. The Veterinary Oncology sector previously evaluated all patients.

### Ultrasound evaluation

Trichotomy of the tumor region was done with up to two centimeters of the peritumoral region. In order to maintain the patient’s comfort during the examination and without sedation or anesthesia, patients were positioned in decubitus according to the anatomical location of the neoplasms. ACUSSON S2000™ equipment (Siemens®, Munich, Germany) was used for all the techniques performed, with a linear transducer and frequency ranging from 8 to 10Mhz. In addition, an ultrasonographic conductive gel was used throughout the examination.

### B-mode ultrasound

The transducer was positioned in the central superficial region of the neoplasms, adjusting the focus, gain, and depth as needed. After adjusting the device, the nodules and masses were measured in longitudinal (length and height) and transversal (width) sections. The characteristics of echogenicity (hypoechogenic or hyperechogenic), echotexture (homogeneous or heterogeneous), echotexture pattern (coarse or smooth), invasiveness in adjacent tissues (presence or absence), capsule (presence or absence), cavitary areas (presence or absence), and hyperechogenic points (presence or absence) were evaluated.

### Doppler

The color Doppler function was activated to identify neovascularization, and the pulse repetition frequency (PRF) was adjusted to 977 Hz. When necessary, changes were made to the pre-established PRF. Tumor neovascularization was characterized according to its intensity (absent, mild, moderate, or intense), location (central, peripheral, or diffuse), and pattern (perinodular, mosaic, or network).

The pulsed wave Doppler was activated and used only for those neoplasms that presented vascularization at color Doppler examination. At this stage, the PRF used in the qualitative assessment was maintained, and the caliper was adjusted to cover 2/3 of the vessel’s caliber, and using an angulation towards the vessel when necessary, respecting the limit of 60° degrees. At least three spectral traces were obtained [14] to get the peak values ​​of systolic velocity (m/s), diastolic velocity (m/s), resistivity index (RI), and pulsatility index (PI).

### ARFI Elastography

The elastographic evaluation was performed using the VTIQ method (virtual touch tissue imaging quantification, 2D-SWE technique). Color elastograms were performed in the qualitative study. Where blue colors represented more elastic areas, green and yellow represented intermediate stiffness, and red corresponded to more rigid areas. Based on the color pattern, neoplasms were classified according to their deformability (deformable or non-deformable). The same elastograms were used for quantitative analysis, and at least three areas of interest (ROIs) were selected. The number of ROIs varied according to the size of the neoplasm, that is, the larger the structure, the more ROIs were measured. The choice of these areas was made to cover both the most rigid and most elastic regions, aiming to obtain a more reliable total representative value. Those areas were randomly chosen to obtain the mean shear wave velocity (SWV - m/s), quantified by the VTIQ software, and using total stiffness as a representative value [14].

### Histopathological evaluation

After ultrasound examinations, clinical care was continued, and biopsies (incisional or excisional) were performed to obtain the definitive diagnosis. Patients were individually anesthetized, and surgical protocols were defined under the recommendation of the responsible veterinarian. These tumor samples were fixed in 10% formalin and sent to the veterinary pathology laboratory within the university, where histological cuts were performed to make slides stained with hematoxylin and eosin and, in cases of mast cell tumors, with toluidine blue. After histopathological diagnosis, neoplasms were classified as benign or malignant, as established by the World Health Organization (WHO).

### Statistical analysis

All data were analyzed using the SPSS Statistics 20 package (IBM®, New York, United States), and a significance level of 95% was used for all tests (*P* < 0.05). Echogenicity, echotexture, texture pattern, invasiveness, capsule, hyperechogenic spots, cavitary areas, and deformability were associated with malignancy using the Chi-square test, and sensitivity, specificity, accuracy, and positive (PPV) and negative (NPV) predictive values ​​were calculated. Logistic regression was performed to differentiate malignancy according to the intensity, location, and pattern of vascularization. The other characteristics were submitted to the Kolmogorov-Smirnov normality test. The Mann-Whitney test was performed to analyze length, width, height, systolic peak, diastolic velocity, and pulsatility index. While for the resistivity index and SWV, a t-test was performed for independent samples. A ROC curve was used to obtain the cut-off point, sensitivity, specificity, and area under the curve for significant results.

Afterward, the variables with significant results were selected, and a descriptive analysis of the association between the different ultrasound techniques was performed. Furthermore, they were grouped into four groups: 1) presence of at least four predictive malignancy characteristics; 2) at least five characteristics; 3) at least six characteristics; 4) seven characteristics. Thus, the chi-square test verified an association with malignancy, and the values ​​of sensitivity, specificity, accuracy, PPV, and NPV were calculated. Additionally, descriptive analysis was performed and expressed in percentages of the qualitative ultrasonographic characteristics and the mean and standard deviation of the quantitative characteristics for each tumor type included in this study, except for single cases.

## Supplementary Information

**Additional file 1.**

## Data Availability

The datasets used and/or analyzed during the current study available from the corresponding author on reasonable request.
